# Endotoxin from Biomass Burning: An Underestimated Health Hazard?

**DOI:** 10.1289/ehp.118-a304b

**Published:** 2010-07

**Authors:** Harvey Black

**Affiliations:** **Harvey Black** of Madison, WI, has written for *EHP* since 1994. His work has also appeared in *Environmental Science & Technology*, *ChemMatters*, and the *Milwaukee Journal Sentinel.*

Approximately 3 billion people worldwide burn biomass—wood, charcoal, dried animal dung, and crop residue—to heat their homes and cook their food. Biomass often is burned in small, poorly ventilated areas; the resulting smoke exposure frequently causes respiratory infections, primarily among women and children younger than 5 years, who spend the most time around the home fires. Recent findings suggest airborne endotoxin generated from burning biomass may play an important role in the health effects associated with biomass smoke **[*EHP*** **118(7):988–991; Semple et al.].**

According to the World Health Organization, exposure to smoke from biomass burning is responsible for 1.5 million premature deaths annually. Previous research has focused primarily on the mass of airborne fine particulate matter as being responsible for the morbidity and mortality caused by biomass burning. These particles can penetrate deep into the lungs, causing inflammation and both acute and chronic airway and lung damage.

Endotoxins are part of the cell wall of gram-negative bacteria and are found in organic material. These molecules can cause lung inflammation and have previously been found in tobacco smoke and in homes where there are pets and mold.

To evaluate the presence of airborne endotoxin in homes where biomass is burned, the researchers set up air sampling monitors in 31 homes in Nepal and 38 homes in Malawi. Average levels of inhalable endotoxin measured over 24 hours in Malawian homes were 24 endotoxin units (EU)/m^3^ for charcoal-burning homes and 40 EU/m^3^ for wood-burning homes. In Nepal, short-term measurements during cooking indicated average inhalable endotoxin levels of 365 EU/m^3^ for dung-burning homes and 43 EU/m^3^ for wood-burning homes. These figures are considerably higher than levels shown to be associated with respiratory ailments during the first two years of life in a separate study [*EHP* 114(4):610–614 (2006)].

The authors acknowledge weaknesses in their study, such as the large time gap between collection of the filters used to trap endotoxins and their analysis, which could have led to high levels of contamination on some of the materials used for collection. Despite the lack of resolution about how biomass smoke contributes to respiratory disease, write the authors, the very fact that it does so makes the use of more efficient stoves and better ventilation in homes where biomass is burned “a matter of urgency.”

## Figures and Tables

**Figure:**
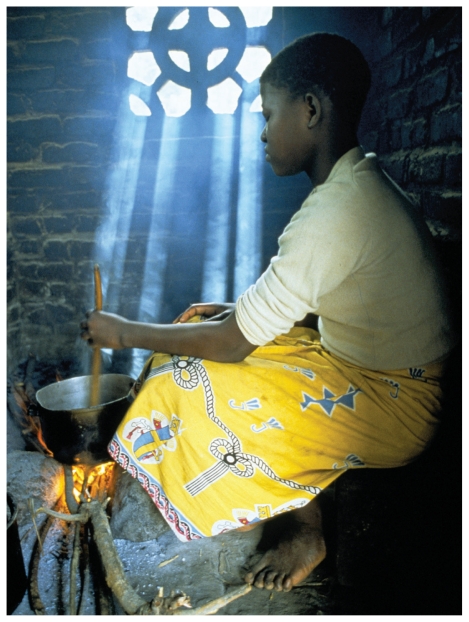
A kitchen in Malawi

